# Higher abundance of DLD protein in buffalo bull spermatozoa causes elevated ROS production leading to early sperm capacitation and reduction in fertilizing ability

**DOI:** 10.1186/s40104-024-01085-6

**Published:** 2024-09-11

**Authors:** Seema Karanwal, Ankit Pal, Fanny Josan, Aditya Patel, Jatinder Singh Chera, Sonam Yadav, Vikrant Gaur, Preeti Verma, Shiva Badrhan, Vitika Chauhan, Mukesh Bhakat, Tirtha Kumar Datta, Rakesh Kumar

**Affiliations:** 1 Animal Genomics Laboratory, Animal Biotechnology Division, ICAR-National Dairy Research Institute, Karnal, Haryana India; 2 NBGC Laboratory, National Bureau of Animal Genetics Resources, Karnal, Haryana India; 3 Artificial Breeding Research Centre, LPM Division, ICAR-National Dairy Research Institute, Karnal, Haryana India; 4 ICAR-Central Institute of Research On Goat, Makhdoom, Mathura, Uttar Pradesh India; 5 ICAR-Central Institute for Research On Buffaloes, Hisar, Haryana India

**Keywords:** Acrosome reaction, Capacitation, High fertile bull, Low fertile bull, Protein, Reactive oxygen species, Spermatozoa

## Abstract

**Backgroud:**

Before fertilization, spermatozoa undergo a crucial maturation step called capacitation, which is a unique event regulates the sperm’s ability for successful fertilization. The capacitation process takes place as the spermatozoa pass through the female reproductive tract (FRT). Dihydrolipoamide dehydrogenase (DLD) protein is a post-pyruvate metabolic enzyme, exhibiting reactive oxygen species (ROS) production which causes capacitation. Additionally, other vital functions of DLD in buffalo spermatozoa are hyperactivation and acrosome reaction. DLD produces the optimum amount of ROS required to induce capacitation process in FRT. Depending on physiological or pathophysiological conditions, DLD can either enhance or attenuate the production of reactive oxygen species (ROS). Aim of this study was to investigate whether changes in the production of ROS in sperm cells can impact their ability to fertilize by triggering the capacitation and acrosome reaction.

**Results:**

In this study, abundance of DLD protein was quantified between high (*n* = 5) and low fertile bull (*n* = 5) spermatozoa. It was found that compared to high-fertile (HF) bulls, low-fertile (LF) bulls exhibited significantly (*P* < 0.05) higher DLD abundances. Herein, we optimised the MICA concentration to inhibit DLD function, spermatozoa were treated with MICA in time (0, 1, 2, 3, 4, and 5 h) and concentrations (1, 2.5, 5, and 10 mmol/L) dependent manner. Maximum DLD inhibition was found to be at 4 h in 10 mmol/L MICA concentration, which was used for further experimentation in HF and LF. Based on DLD inhibition it was seen that LF bull spermatozoa exhibited significantly (*P* < 0.05) higher ROS production and acrosome reaction in comparison to the HF bull spermatozoa. The kinematic parameters of the spermatozoa such as percent total motility, velocity parameters (VCL, VSL, and VAP) and other parameters (BCF, STR, and LIN) were also decreased in MICA treated spermatozoa in comparison to the control (capacitated) spermatozoa.

**Conclusions:**

The present study provides an initial evidence explaining the buffalo bull spermatozoa with higher DLD abundance undergo early capacitation, which subsequently reduces their capacity to fertilize.

**Supplementary Information:**

The online version contains supplementary material available at 10.1186/s40104-024-01085-6.

## Background

Fertilization is a complex biological process that remains not fully understood in many aspects. Success or failure of fertilization is determined by several sperm and egg factors [1]. For effective mammalian fertilization, sperm capacitation is an essential process. Sperm "capacitation", initially identified in the 1950s by Austin [2] and the biochemical process occurs in the female reproductive tract, which enables sperm to fertilize an ovum. The process of capacitation has been identified as a complex phenomenon over time, involving changes in the spermatozoa’s intracellular pH, intracellular reactive oxygen species (ROS), intracellular calcium levels, intracellular cAMP levels, and plasma membrane fluidity [3, 4]. These cellular modifications during capacitation results in three physiological changes: (a) hyperactivation (increased motility); (b) tyrosine phosphorylation in several kinds of proteins; and (c) acrosome reaction (expulsion of the acrosomal contents) [5–7]. The initial two events indicate a temporal connection with capacitation, while the acrosome reaction appears to be the capacitation’s end point [7]. Hyperactivated spermatozoa are thought to be able to cross the female reproductive tract’s mucosal barrier because of their increased motility. Furthermore, a variety of proteins have been found to undergo tyrosine phosphorylation in several species during capacitation [6–12], such as A kinase anchoring proteins have been identified as capacitation-dependent phosphorylated proteins in human [12], mouse [13], rat [14], and hamster [15] spermatozoa; however, very few of these proteins identified so far. Other proteins, namely heat shock protein 90 [16], calcium-binding tyrosine phosphorylation-regulated protein [17], metabolic enzymes like dihydrolipoamide dehydrogenase (DLD) [18, 19] and pyruvate dehydrogenase A (PDHA) [19] have also been found to get tyrosine phosphorylated in a capacitation-dependent manner in mouse spermatozoa. The capacitation process culminates in the acrosome reaction, which begins when the spermatozoon’s plasma membrane fuses with the outer acrosomal membrane, creating fused membrane vesicles resultantly releasing their molecular contents. The acrosome reaction is an essential step in the fertilization process since the released acrosomal contents have been associated with oocyte zona pellucida penetration [20].

**Fig. 1:**
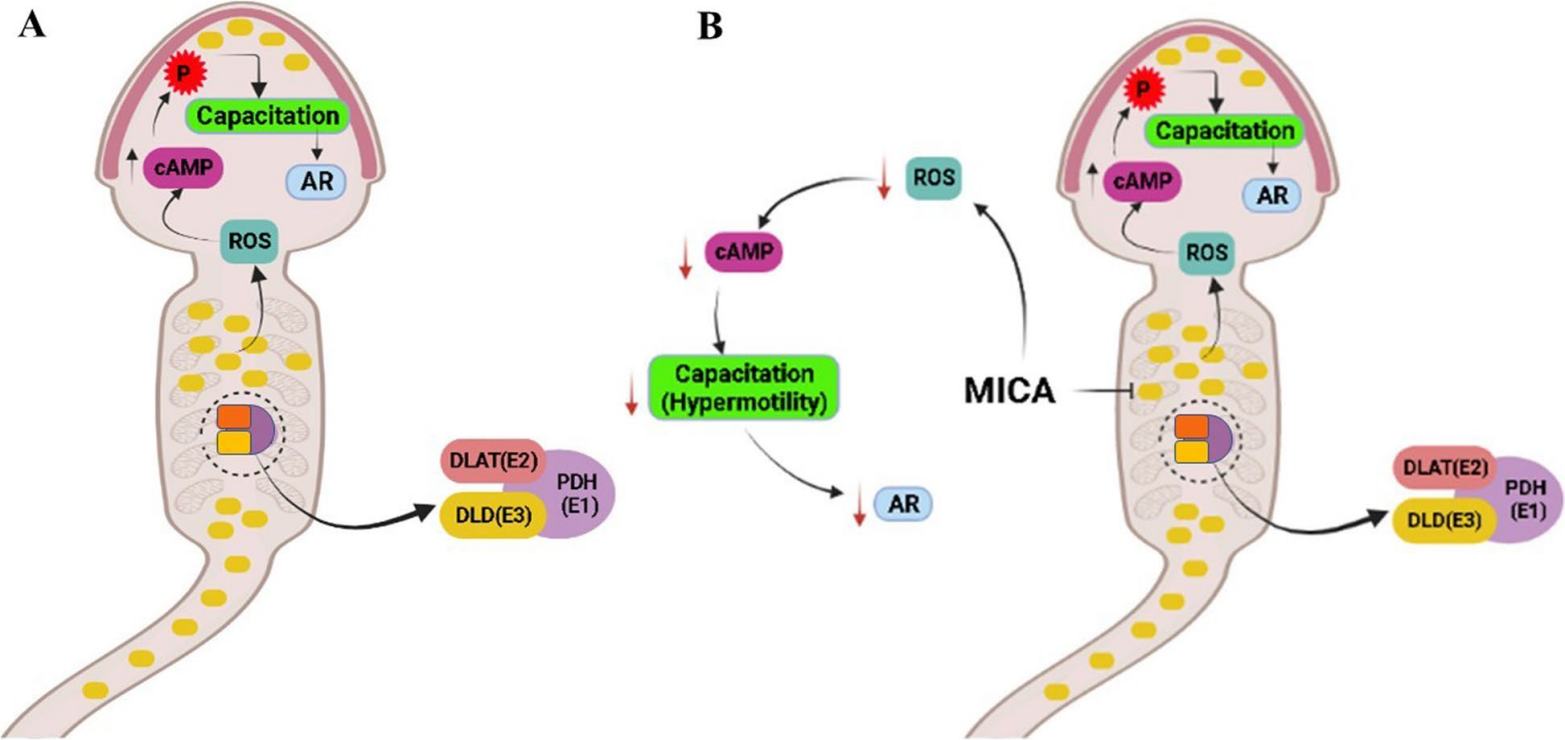
Graphical representation of DLD protein signal pathway in buffalo sperm. **A** Proposed model of the DLD protein signal pathway in buffalo sperm to elicit the function. In the normal condition DLD protein present in the mitochondria bind to the PDH complex. DLD protein produces optimum amount of ROS to induce capacitation in the spermatozoa. ROS production increases the cAMP level which in turn phosphorylate the proteins, this condition induces capacitation or hypermotility. Capacitation leads to the acrosome reaction that help in the fertilization process. **B** MICA induced DLD protein inhibition in buffalo sperm. MICA inhibits the DLD protein, this would result in a reduction in ROS levels, which will, in turn, result in a reduction in cAMP levels with a decline in capacitation or acrosome reaction

Although it is well known that capacitation is essential for successful fertilization process, but what happens if spermatozoa are capacitated before they get to the fertilisation site? In our previous study using Westen blot analysis, we reported that the abundance of DLD was significantly higher in low fertile bull sperm as compared to high fertile [21]. DLD is a redox enzyme found in the mitochondria, involved in decarboxylation of pyruvate to form acetyl-CoA during the cascade of glucose metabolism and mitochondrial adenine triphosphate (ATP) production. Action of DLD involved in the process of capacitation and acrosome reaction by regulating sperm intracellular lactate, intracellular pH, and intracellular calcium [18, 22–24]. Inhibition of DLD was achieved by the use of the DLD-specific inhibitor, 5-methoxyindole-2-carboxylic acid (MICA), inhibit capacitation and acrosome reaction in hamster spermatozoa [25]. This happens because MICA treated spermatozoa showed lactate accumulation (due to pyruvate dehydrogenase complex (PDHc)/DLD inhibition and thus, pyruvate nonconsumption), which resulted in lowering of initially, the intracellular pH and eventually, the intracellular calcium leads to blocked capacitation and acrosome reaction [24, 26]. Depending on physiological or pathophysiological conditions, DLD protein can either enhance or attenuate the production of ROS [26]. Based on the previous research it was found that in spermatozoa DLD produce optimum amount of ROS required to induce capacitation process in FRT [27, 28]. Since DLD involved in the ROS production, herein we hypothesized that the spermatozoa of high and low fertility bulls contain differential abundance of DLD proteins that may induce differential ROS, therefore, this differential ROS can affect the capacitation and acrosome reaction of spermatozoa, ultimately curtailing the fertilizing potential of the bulls (Fig. 1). The main objective of this study was to quantify differential abundance of DLD protein in high and low fertile bull spermatozoa and how the fertilization capacity of sperm can be impacted by the increased level of DLD protein in buffalo spermatozoa. The current study helps in better understanding of DLD protein in manoeuvring sperm capacitation and acrosome reaction which in turn reflect on reduced fertilizing ability of the spermatozoa.

## Materials and methods

All chemicals, media and reagents used in this study were obtained from Sigma-Aldrich India, Thermo Fisher Scientific and Invitrogen, USA. All plastic ware was purchased from Nunc (Thermo Fisher Scientific, USA). The instruments used in the study are as follows: BD Accuri C6 flow cytometry (Becton Dickinson Biosciences, Ann Arbor, MI, USA with BD Accuri C6 flow software v.1.0.27.1), CO_2_ incubator (Innova-Co 170; New Brunswick Scientific, Edison, NJ, USA), Stereo zoom microscope (Nikon smz1000), Fluorescence microscope (Olympus Scientific, Tokyo, Japan) and Centrifuge (Z326K, Hermle Labortechnink, Wehngen, Germany).

### Buffalo bull classification and semen straws collection

The classification of high and low fertile bulls was established based on their conception rate. Eighty-two Murrah buffalo bulls were selected through a progeny testing program after assessing their breeding soundness and semen quality parameters, including sperm count, viability, and progressive motility. These bulls were kept under a regular feeding and management schedule. The statistical methodology, which is extensively described in our prior study [21], was utilized to determine the conception rate (CR) of each bull. In brief, CRs of the 82 bulls considered in this study were subjected to Shapiro–Wilk normality test and they were found to fit into normal distribution (*P*-value = 0.781; where the null hypothesis of data fitting into a normal distribution was accepted) with a mean of 43.06% and a standard deviation (SD) of 5.05%. Thus, average fertile bulls were identified as those whose CRs ranged between 48.11% and 38.01%. Ten buffalo bulls (*Bubalus bubalis*, *n* = 10), five each in high-fertility (CR between 51% to 56.7%) and low fertility (CR between 28.8% to 33.8%) chosen for the study had their CRs above and below the Mean ± 1 SD (HF > 48.11% and LF < 38.01%). The cryopreserved semen straws of each bull were obtained from National Dairy Research Institute (NDRI) and Central Institute for Research on Buffaloes (CIRB).

### Quantitation of DLD proteins in high and low fertile bulls

The cryopreserved semen was pooled from each high (*n* = 5) and low fertile bulls (*n* = 5) separately and pre-processing was performed. For pre-processing 1 × PBS was added to the semen samples and centrifuged (700 × *g*) at 4 °C for 10 min. The sperm pellet was resuspended in 100 μL PBS and counted in flow cytometry. An equal number of spermatozoa from high and low fertile were taken for further experimentation. To quantify the DLD proteins, 10 million spermatozoa were used to prepared smear on a poly-L-lysine coated slide (Sigma-Aldrich). The spermatozoa were fixed using paraformaldehyde (4% in 1 × PBS) at 4 °C for 20 min. Excess paraformaldehyde was rinsed off with PBS and then the sperms were permeabilized with permeabilization buffer (0.1% Triton X-100 in PBS) for 15 min at room temperature because the target protein was intracellular. The excess amount of permeabilization buffer was washed with PBS. The cells were incubated with 2% bovine serum albumin (BSA) for 1 h at room temperature to block the unspecific binding of the antibodies. Then slides were overlaid with a solution containing the primary antibody DLD (1:500) in blocking buffer (2% blocking buffer in 1 × PBS) and incubated for 14 h at 4 °C in the dark. After incubation the solution was decanted, and the cells were washed three times each for 5 min in 1 × PBST (1 × PBS with 0.1% Tween 20). FITC labelled secondary antibody goat anti-rabbit DLD (1:500) (catalogue No. ab97050, Abcam) were added in 2% BSA for 1 h at room temperature in the dark. Then, the slides were washed twice in PBS-T and counter staining was performed with 0.1 μg/mL DAPI (catalogue No. D9642, DNA stain) for 1 min and rinsed with 1 × PBST. Coverslip was mounted with a drop of DABCO to visualized the cells under fluorescence microscope (Olympus IX73, Tokyo, Japan). The micrographs were afterwards used for fluorescence quantification analysis using the ImageJ software. The fluorescent regions were identified and quantified for a minimum of 200 spermatozoa in at least 10 fields covering the entire slide (pooled sample of biological replicates × 3 technical replicates). The region of interest (ROI) on the individual spermatozoon was selected according to the antibody binding pattern, e.g., the whole sperm periphery region was considered as the ROI for DLD. The fluorescence intensities were quantified at random from the micrographs that were captured using similar acquisition settings, i.e., exposure time, field area and magnification etc. The raw integrated density values for the chosen ROIs were later normalized and these values were used to obtain the mean fluorescent intensity (MFI) values used in subsequent experiments.

### Evaluation of in vitro capacitation using CTC assay

**Fig. 2:**
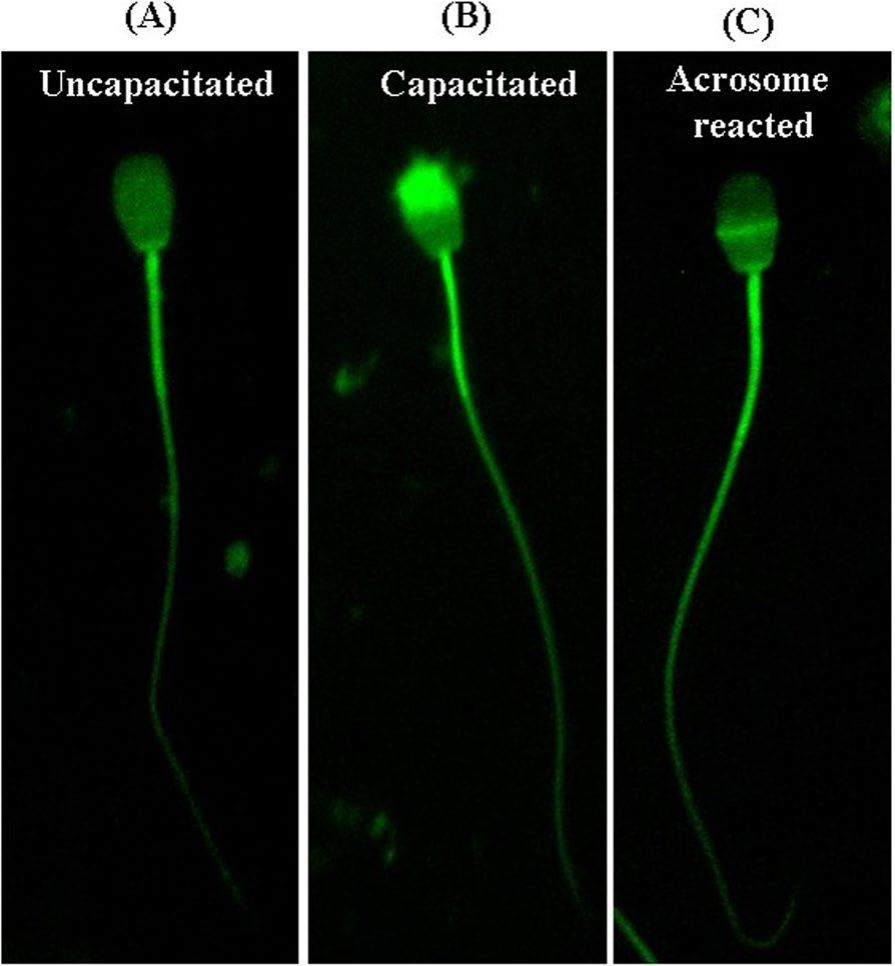
The figure is illustrating induced capacitation in spermatozoa using CTC staining. **A** Non-capacitated-Bright fluorescence over the entire sperm head. **B** Capacitated-Prominent fluorescent positive acrosome and fluorescence-free (dark) band in the post-acrosomal region. **C** Acrosome-reacted-Sperm exhibits low fluorescent signal throughout the sperm head, with remaining positive signal in the equatorial segment and mid-piece

In vitro capacitation of the buffalo bull spermatozoa was induced by suspending the pre-processed spermatozoa (as mentioned above) in the capacitating medium (Sp-TALP containing 6 mg/mL bovine serum albumin (BSA), 2 mmol/L CaCl_2_•2H_2_O and 10 mmol/L NaHCO_3_). The semen samples were kept in capacitating medium for 6 h in 5% CO_2_ incubator (Innova-Co 170; New Brunswick Scientific, Edison, NJ, USA), at 37 °C. The sperm capacitation was confirmed by chlortetracycline (CTC) staining. Briefly, pre-processed spermatozoa were incubated with 40 μL of CTC working stain (containing 750 mmol/L CTC, 5 mmol/L L-cysteine, 130 mmol/L NaCl, and 20 mmol/L Tris–HCl, pH 7.8) in 1.5-mL microcentrifuge tube (MCT) at 37 °C for 20 min in dark. Later, 3.2 μL of 4% paraformaldehyde (PFA) was added and incubated for 5 min [28]. The samples were then washed, smeared on glass slides, mounted with one drop of DABCO R 33-LV then observed under fluorescence microscope (Olympus IX73, Tokyo, Japan) using UV filter. Acrosomal staining pattern of spermatozoa was classified as follows: (A) uncapacitated sperm have bright fluorescence over the entire sperm head, mid-piece and intact acrosome, (B) capacitated sperm have prominent fluorescent at equatorial segment, mid-piece and fluorescence-free (dark) band in the post-acrosomal region with intact acrosome, and (C) acrosome reacted sperm have low fluorescent signal in sperm head, bright fluorescence in the equatorial segment and mid-piece (Fig. 2). Sperm with a nonspecific or intermediate fluorescent signal status were not selected for subsequent analysis.

### Standardisation of MICA concentration to inhibit DLD protein in spermatozoa

DLD protein can be inhibited by MICA (5-Methoxyindole-2-carboxylic acid) (catalogue No. M14951, Sigma Aldrich, USA). The DLD protein generated the optimum amount of ROS that induced capacitation. To optimize the MICA concentration, we thus assess the reduction in ROS generation following DLD inhibition. Here we used different concentrations of MICA such as 1, 2.5, 5, and 10 mmol/L at 0, 1, 2, 3, 4, and 5 h time intervals. After inhibition of DLD protein we assessed reduction in ROS production by staining the spermatozoa using MitoSOX™ Mitochondrial Superoxide Indicators, for live-cell imaging (catalogue No. M36008, Thermo Fisher Scientific and Invitrogen, USA). The production of superoxide in mitochondria was analysed by flow cytometry using MitoSOX superoxide indicator.

Briefly, the random bull spermatozoa were counted using flow cytometry. In each tube 0.2 million spermatozoa were added with different concentrations of MICA containing capacitating media and incubated for different time interval (0–5 h) at 38.5 °C in a CO_2_ incubator. Following the MICA treatment, an approximately 200 μL 1 × Sp-TALP was added to each tube and tubes were subjected to centrifugation at 700 × *g* to remove traces of MICA inhibitor at 37 °C. Supernatant were removed from each tube and pellet resuspended in 40 μL 1 × Sp-TALP. Then 13 μL working MitoSOX red (5 μmol/L) were added to each tube and incubated for 30 min. The traces of MitoSOX red were removed by centrifugation. The pellet was resuspended in 100 μL Sp-TALP and subjected to flow cytometry. For every experiment, three technical replicate samples were prepared. As a routine SOP of the equipment, quality control (QC) of flow cytometer was performed prior to each experiment. It guarantees that the flow cytometer is functioning properly and generating consistent, accurate, and repeatable data. The performance of the instrument was routinely monitored and calibrated using reference particles or beads with known qualities as part of QC. Flow cytometry was performed with a standard bench-top BD Accuri C6 flow cytometer (Becton Dickinson Biosciences, Ann Arbor, MI, USA, with BD Accuri C6 software v.1.0.27.1). The machine was calibrated daily according to the manufacturer’s recommendations with 8 and 6 peak calibration beads, and the QC was performed every second day using BD CS &T RUO beads. The 488-nm laser was used for the excitation of FITC, and its emission was filtered using a 533/30 bandpass filter. The filtered emissions were detected by photomultiplier tubes. A threshold of 80,000 in the forward scatter (FSC) signal was applied to remove the electrical noise and very small events. The samples were collected at the default low flow rate (14 µL/min). For each sample, 10,000 individual events were acquired. The cell population of events in FSC-SSC dot plots was identified and used for the data analysis. No compensation or voltage gain settings was applicable. Further analysis was done using BD FlowJo software v10.6.1 and the MIFlowCyt guidelines were followed to the maximum extent possible.

### Evaluation of ROS production in high and low fertile bull spermatozoa after DLD inhibition

To evaluate the ROS production after DLD inhibition in high and low fertile bull spermatozoa, 0.2 million spermatozoa were collected from each group. The optimized concentration of MICA (10 mmol/L) was added to the tube containing spermatozoa from each group and incubated for 4 h at 38.5 °C in a CO_2_ incubator. Then 200 µL 1 × Sp-TALP was added to the samples followed by centrifugation at 700 × *g* for 5 min to wash the samples. The working MitoSOX red (5 μmol/L) was added to washed samples and incubated for 30 min. Approximately, 200 µL 1 × Sp-TALP was added to wash the sample and then centrifuged at 700 × *g* for 5 min. The supernatant was removed and sample was resuspended in 100 µL 1 × Sp-TALP. All the samples were prepared in triplicate and the change was analysed in flow cytometry.

### Evaluation of acrosome reaction in high and low fertile bull spermatozoa after DLD inhibition

To evaluate the acrosome reaction in high and low fertile bull spermatozoa after DLD inhibition, 0.2 million processed spermatozoa were added to the MCTs from each group. The sample were treated with 10 mmol/L MICA inhibitor and incubated for 4 h at 38.5 °C in a CO_2_ incubator. After treatment approximately, 200 µL 1 × Sp-TALP was added to the samples and centrifuged at 700 × *g* for 5 min to remove the traces of MICA. To determine the acrosome status of MICA treated spermatozoa, fluorescein isothiocyanate conjugated peanut agglutinin (FITC-PNA; 25 μg/mL) (λ_ex_ = 494 nm and λ_em_ at 517 nm) was added followed by 15 min incubation at 37 °C in the dark [29]. Approximately, 200 µL 1 × Sp-TALP was added to the samples followed by centrifugation at 800 × *g* for 3 min to wash the sample. The supernatant was removed and sample was resuspended in 100 µL 1 × Sp-TALP and change in acrosome reaction was analysed by flow cytometer as mentioned in the earlier section.

### Sperm kinematic parameters analysis after DLD inhibition

The spermatozoa incubated with MICA in capacitated medium were subject to computer assisted sperm analysis (CASA) for estimating the velocity and motion parameters of the buffalo bull spermatozoa. Computer-assisted sperm analyzer (IVOS12.1, Hamilton-Thorne Biosciences, Beverly, MA, USA) was used to evaluate the kinetic characteristics. The motility and movement parameters like the curvilinear velocity (VCL, μm/s), linear velocity (VSL, μm/s), average path velocity (VAP, μm/s), the percentage of linearity, i.e., the ratio between VSL and VCL (LIN, %), the straightness coefficient which is the ratio between VSL and VAP (STR, %) and the frequency with which the actual sperm trajectory crossed the average path trajectory (BCF, Hz) were recorded in triplicates for all the experimental groups. The CASA software settings were as follows: temperature = 38 °C, frame rate = 60 Hz, frames acquired = 30, minimum contrast = 35, minimum cell size = 5 pixels, cell size = 9 pixels, cell intensity = 110 pixels, progressive cells (VAP cut-off = 50 m/s, STR cut-off = 70%), slow cells (VAP cut-off = 30/s and VSL cut-off = 15/s). The spermatozoa (*n* = 500) were observed in a minimum of 5 optical fields around the central reticulum of the chamber for sperm motility analysis. The differential kinematic parameters among the various experimental groups were measured by ANOVA and Tukey’s post-hoc test, as implemented in GraphPad Prism 9.0 (for Windows, GraphPad Software, La Jolla, California, USA, www.graphpad.com) and *P* < 0.05 was considered to be statistically significant.

### Statistical analysis

Data was analysed by GraphPad prism 9.0 (GraphPad Software, San Diego, CA, USA). Quantitative differences in the MFIs produced upon DLD antibody binding between the HF and LF bull’s spermatozoa were analysed by an unpaired two-tailed *t*-test and *P*-value < 0.05 was considered statistically significant. The impact of DLD inhibition (trough MICA) in ROS production and acrosome reaction in buffalo bull spermatozoa was analysed by two-way ANOVA followed by paired wise comparison of means with Dunnett’s multiple comparisons test. The kinematic parameters after DLD inhibition between treated and control spermatozoa was analysed by two-way ANOVA followed by paired wise comparison of means with Dunnett’s multiple comparisons test. The statistical analysis was done at 95% confidence level (*P* < 0.05) was considered statistically significant. The differential ROS production and acrosome reaction before and after DLD inhibition (with optimised MICA concentration) in HF and LF bulls was analysed by an unpaired two tailed *t*-test. The differential kinematic parameters after DLD inhibition (with optimised MICA concentration) in high and low fertile bulls was analysed by an unpaired two tailed *t*-test and *P* < 0.05 was considered statistically significant.

## Results

### Quantification of fluorescence signal of DLD protein in HF and LF buffalo bull spermatozoa

**Fig. 3:**
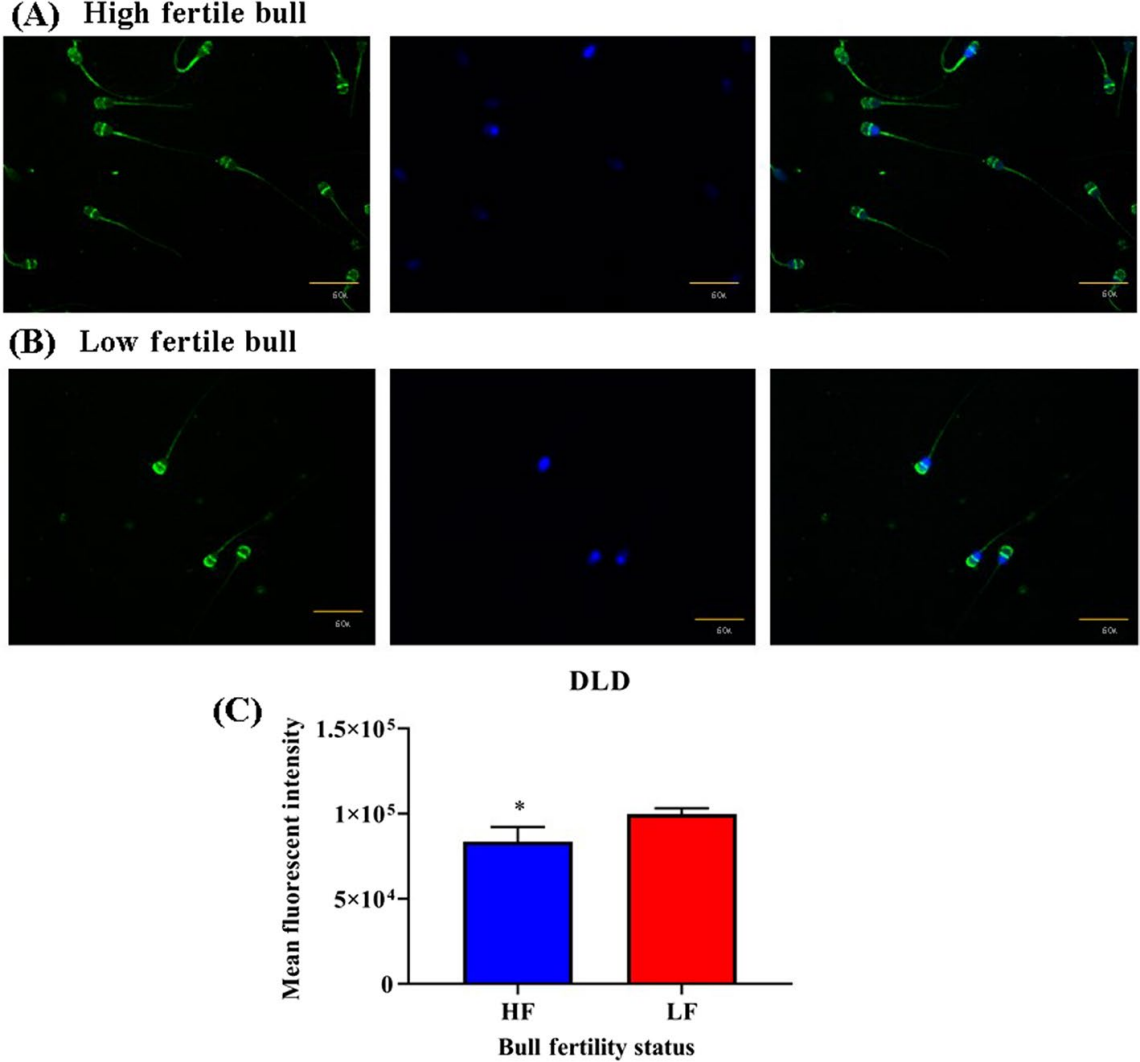
The figure is illustrating localization of DLD protein in HF and LF. **A** High fertile bull spermatozoa. **B** Low fertile bull spermatozoa. **C** Histogram representing average mean fluorescence intensity DLD protein in high and low fertile bull spermatozoa. Where * indicates significant different value *P* < 0.05

To quantify the DLD protein in high and low fertile buffalo bull spermatozoa immunocytochemistry (ICC) was performed. In which approximately 10%–12% of the total buffalo sperm population was found to be unstained, as inferred from the comparison of fluorescent micrographs with the bright-field micrographs. It was found that DLD localized predominantly in the head and in midpiece of spermatozoa of contrasting fertility bulls (Fig. 3A) This unstained sperm-population was excluded from the fluorescence quantification analysis. Quantification of DLD protein revealed significantly difference in mean fluorescent intensities in HF and LF bull spermatozoa. We observed that abundances of DLD protein was significantly (*P* < 0.05) higher in low-fertile bull sperm than that of high fertile bulls (Fig. 3).

### ROS production by spermatozoa after DLD inhibition

**Fig. 4:**
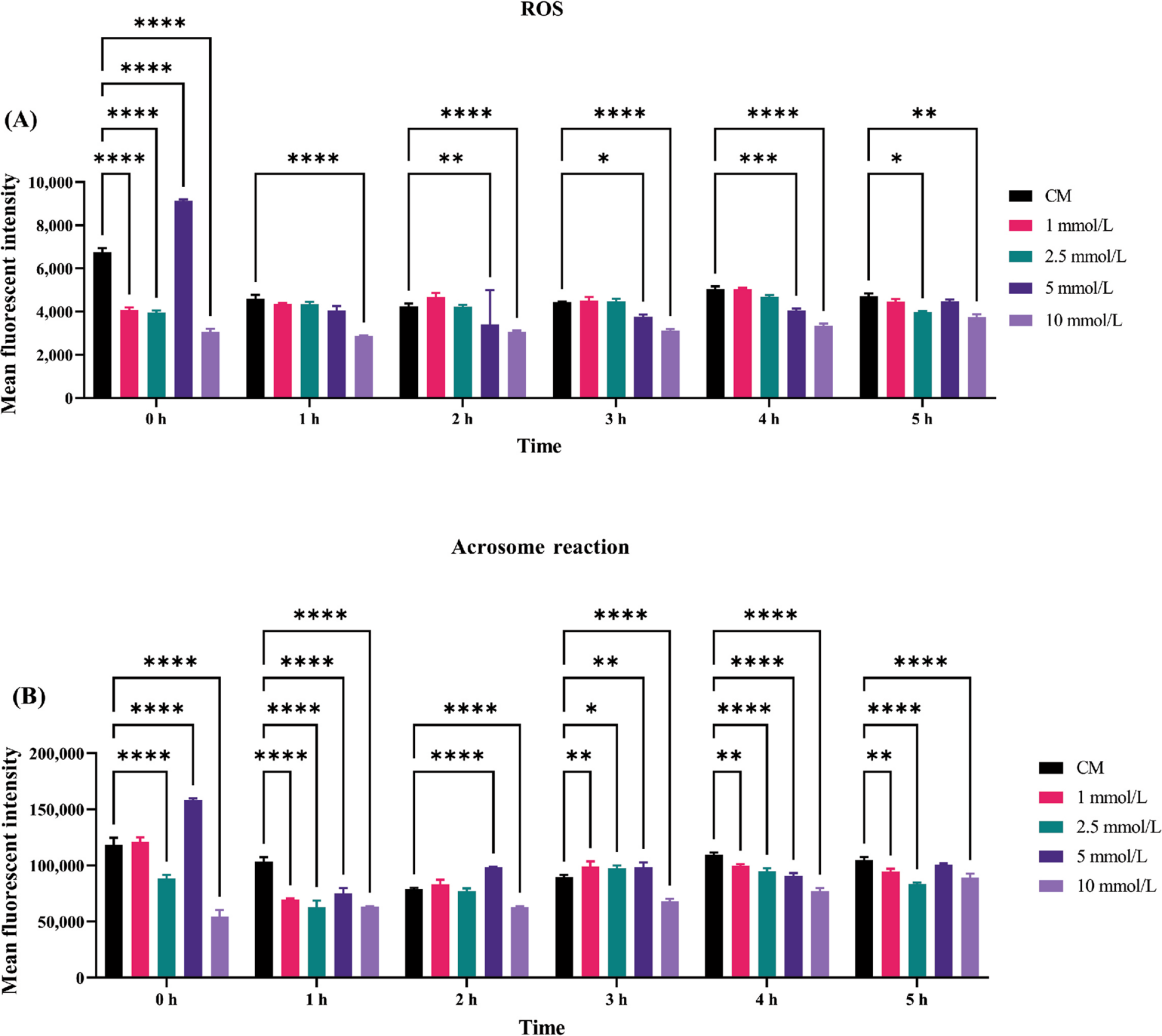
The figure is representing Average MFI histograms of ROS production and AR in random bull spermatozoa after DLD inhibition. **A** Average MFI histograms of ROS production in random bull spermatozoa after DLD inhibition in time (0 h, 1 h, 2 h, 3 h, 4 h, 5 h) and dose (1 mmol/L, 2.5 mmol/L, 5 mmol/L, and 10 mmol/L MICA concentrations) dependent manner. **B** Average MFI histograms of acrosome reaction produced in random bull spermatozoa after DLD inhibition in time (0 h, 1 h, 2 h, 3 h, 4 h, 5 h) and dose (1 mmol/L, 2.5 mmol/L, 5 mmol/L, and 10 mmol/L MICA concentrations). Data represent means ± SEM. * indicates* P* < 0.05, ** P < 0.01, *** P < 0.001 and **** *P* < 0.0001 versus control. Two-way ANOVA analysis were performed

Reactive oxygen species levels were estimated in capacitated buffalo spermatozoa in the presence of MICA to assess the contribution, if any, of DLD to ROS levels (Fig. 4A). ROS production after MICA treated spermatozoa at 0 h with 1, 2.5 and 10 mmol/L concentrations was significantly decreased (*P* < 0.05) compared to the control spermatozoa. In contrast, the 5 mmol/L concentration showed a significant increase (*P* < 0.05) in ROS production compared to the control. At the 1 h 10 mmol/L concentration, it was found that ROS level was significantly decreased (*P* < 0.05) compared to the control spermatozoa. ROS generation was significantly (*P* < 0.05) reduced after 2, 3, and 4 h of incubation in 5 mmol/L MICA treated spermatozoa in comparison to control. Comparable outcome was noted in the spermatozoa treated with 10 mmol/L MICA at 2, 3, and 4 h intervals. Furthermore, reduced ROS are also produced by spermatozoa treated with 10 mmol/L MICA after 2, 3, and 4 h of incubation. Nevertheless, ROS levels in 2.5 and 10 mmol/L MICA treated spermatozoa after 5 h incubation were significantly reduced as compared to the control spermatozoa. Furthermore, compared to other time intervals such as 1, 2, 3, and 4 h at 10 mmol/L MICA concentration, the degree of ROS reduction was found to be lowered after 5 h treatment with 10 mmol/L MICA. Moreover, significant reduction in ROS was observed in 10 mmol/L concentration at all time intervals, however, 4 h at 10 mmol/L has been selected for further experimentation.

### Spermatozoa acrosome reaction after DLD inhibition

Acrosome reaction levels were estimated in capacitated buffalo spermatozoa in the presence of MICA to assess the contribution, if any, of DLD to acrosome reaction levels (Fig. 4B). Acrosome reaction level in MICA treated spermatozoa at 0 h with 2.5 mmol/L and 10 mmol/L concentrations was significantly decreased (*P* < 0.05) compared to the control spermatozoa, however, at the 1 mmol/L acrosome reaction slightly increased but not significantly. In contrary, 5 mmol/L concentration showed a significant increase (*P* < 0.05) in acrosome reaction level compared to the control. It was found that at the 1 and 4 h in all the MICA concentrations (1, 2.5, 5 and 10 mmol/L) acrosome reaction level was significantly decreased (*P* < 0.05) compared to the control spermatozoa. Acrosome reaction level at 2 h found to be significantly (*P* < 0.05) decreased in 2.5 mmol/L and 10 mmol/L concentration with comparison to control spermatozoa. However, contrary, 5 mmol/L concentration showed a significant increase (*P* < 0.05) in acrosome reaction level compared to the control after 2 h treatment. Additionally, it was found that in 1, 2.5, 5 mmol/L after 3 h treatment the acrosome reaction significantly increased in comparison to the control spermatozoa. However, acrosome reaction level significantly decreased in 10 mmol/L at 3 h treatment with respect to control spermatozoa. After the 5 h treatment in 1, 2.5 and 10 mmol/L concentration acrosome reaction decreased significantly. Moreover, the level of acrosome reacted spermatozoa was found to be increased in 5 mmol/L concentration at 5 h treatments but not significantly. As results significant ROS reduction was observed in 10 mmol/L concentration at all time intervals. However, 4 h at 10 mmol/L was selected for further experimentation because this time interval showed a noticeable trend in both ROS production and acrosome reaction.

### Kinematic parameters of spermatozoa after DLD inhibition

**Fig. 5:**
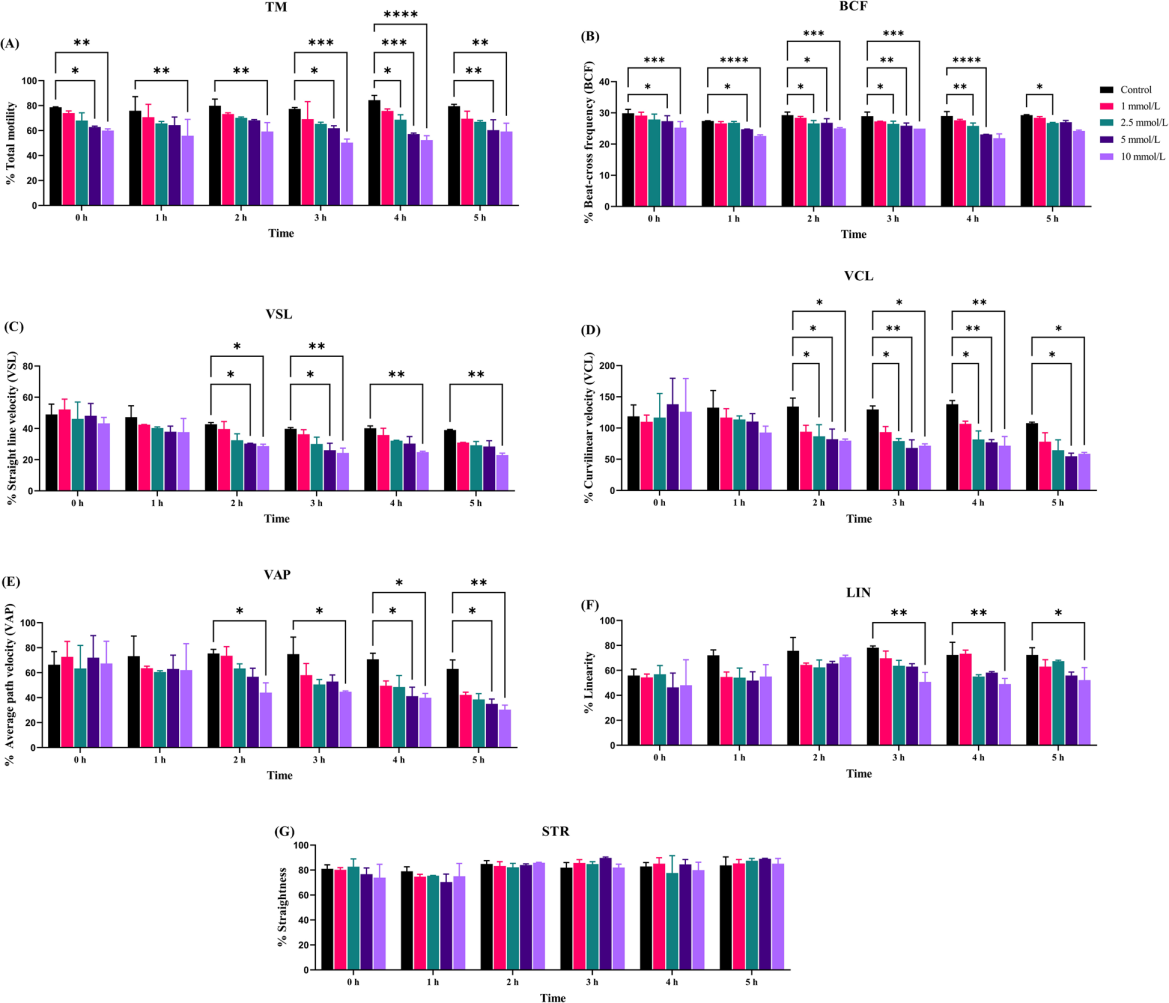
The figure is representing dose and time-dependent effect of MICA on buffalo spermatozoa motility. MICA used in different concentrations, i.e., 1, 2.5, 5, and 10 mmol/L were adjusted throughout a variety of time intervals; 0, 1, 2, 3, 4, and 5 h, sperm samples were washed and subjected to CASA. **A** TM. **B** BCF. **C** VSL. **D** VCL. **E** VAP. **F** LIN. **G** STR. Data represent means ± SEM. * indicates* P* < 0.05, ** P < 0.01, *** P < 0.001, and **** P < 0.0001 versus control. ANOVA analysis was performed

The kinematic parameters of spermatozoa were determined using computer-assisted semen analysis (CASA) following DLD inhibition to find out the function of DLD in hyperactivation. It was discovered that the percent total motility of the MICA treated spermatozoa was significantly reduced with respect to control spermatozoa. The velocity parameters of the treated spermatozoa such as VCL, VSL and VAP were found significantly decreased in comparison with control spermatozoa. The treated spermatozoa also showed a reduction in BCF and LIN, two other CASA measures (Fig. 5A–G). In contrast, no significant difference was observed between treated and control spermatozoa in the STR. The mean of the sperm kinematic parameters is presented in Table S1.

### Differential level of ROS production in HF and LF bull spermatozoa after DLD inhibition

**Fig. 6:**
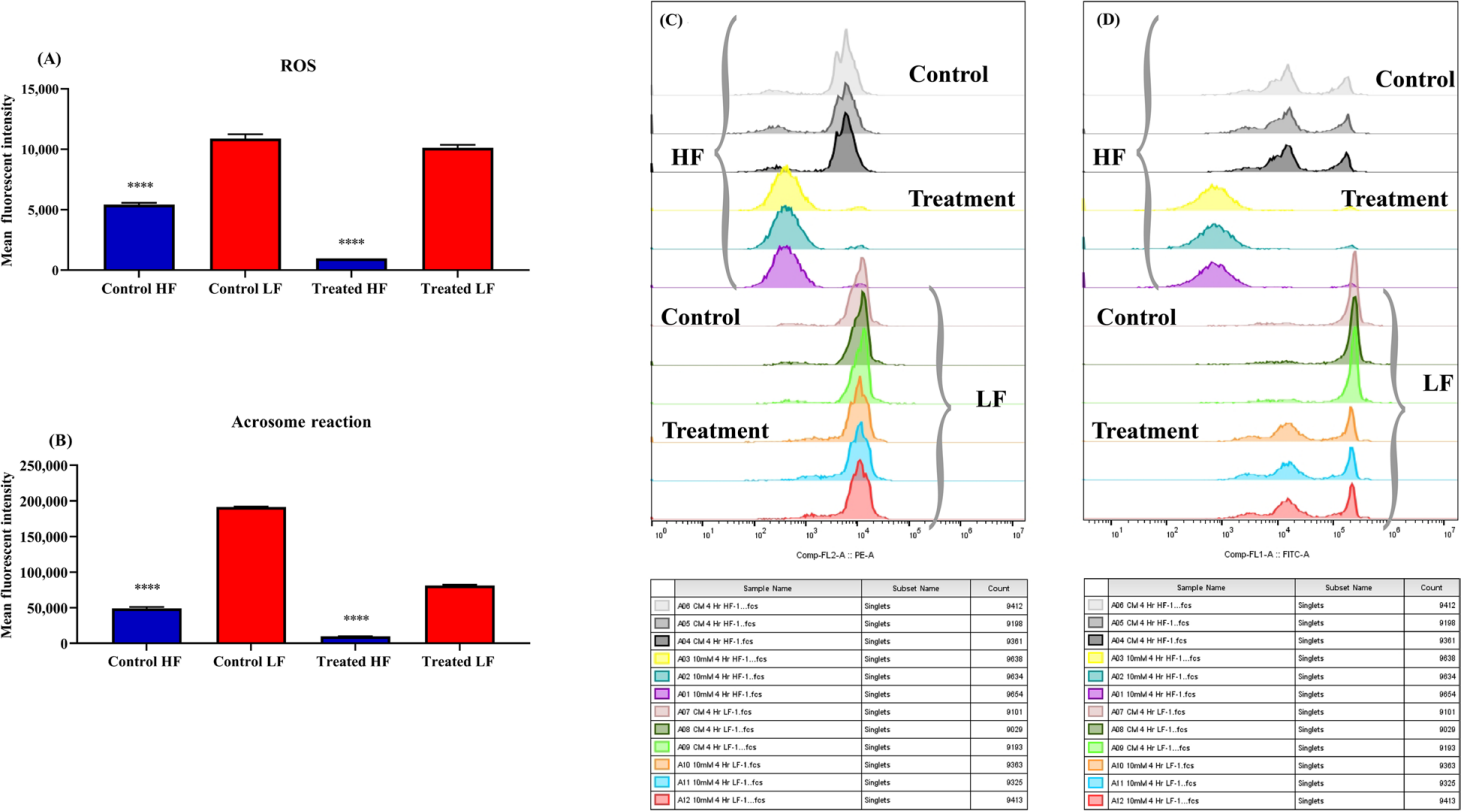
The figure is representing ROS and AR in HF and LF buffalo bull spermatozoa after DLD inhibition. **A** Average MFI histograms of ROS produced in control (capacitated) HF versus LF bull spermatozoa and MICA treated HF versus LF bull spermatozoa. **B** Average MFI histograms of acrosome reaction in control (capacitated) HF versus LF bull spermatozoa and MICA treated HF versus LF bull spermatozoa HF versus and LF bull spermatozoa. **C** Overlay MFI histograms of ROS generation obtained by flow cytometry analysis of spermatozoa from bulls of high and low fertile bulls (*n* = 5, each group). **D** Overlay MFI histograms of acrosome reaction obtained by flow cytometry analysis of spermatozoa from bulls of high and low fertile bulls (*n* = 5, each group). Data represent means ± SEM. **** indicates* P* < 0.0001 versus respective control, ANOVA analysis was performed

To confirm the differential levels of ROS generation upon DLD inhibition, the spermatozoa from HF and LF buffalo bulls were subsequently subjected to flow cytometry. It was discovered that, in comparison to control (capacitated) LF bull spermatozoa, the ROS generation in the control HF bull was significantly reduced (Fig. 6A). Furthermore, following MICA treatment, it was found that the HF bull spermatozoa generated substantially less ROS than the LF bull spermatozoa (Fig. 6A and C). In addition, the treated HF spermatozoa produce reduced ROS versus the control HF spermatozoa; the LF bull spermatozoa exhibit similar results (Additional file 1: Fig. S2A and C).

### Differential level of acrosome reaction in HF and LF bull spermatozoa after DLD inhibition

To confirm the different levels of acrosome reaction upon DLD inhibition, the spermatozoa from HF and LF buffalo bulls were subsequently subjected to flow cytometry. It was discovered that, in comparison to control (capacitated) LF bull spermatozoa, the acrosome reaction in the HF bull control was significantly reduced (Fig. 6B). Additionally, it was discovered that the acrosome reaction after MICA treatment in HF bull spermatozoa was significantly reduced than that of the LF bull spermatozoa (Fig. 6B and D). Furthermore, the treated HF spermatozoa exhibit a decreased acrosome reaction in comparison to the control HF spermatozoa; same results have been observed in the LF bull spermatozoa (Additional file 1: Fig. S2B and D).

### Kinematic parameters of HF and LF bull spermatozoa after DLD inhibition

**Fig. 7:**
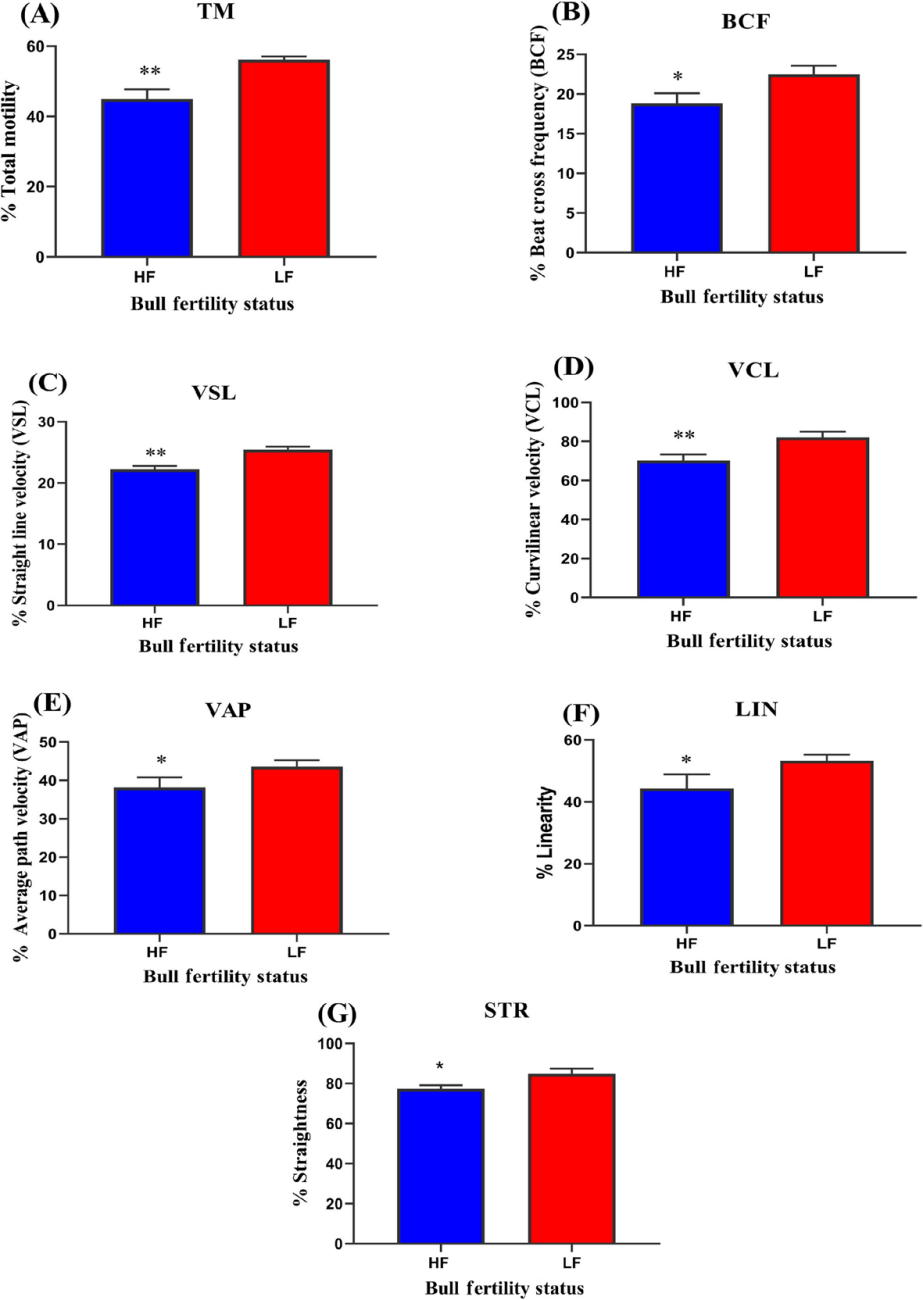
The figure is representing kinematic parameter of HF and LF buffalo bull spermatozoa after DLD inhibition. High and low fertile bull spermatozoa were treated to preoptimized MICA concentration (10 mmol/L of MICA for 4 h). The samples were pre-processed and subjected to CASA. **A** TM. **B** BCF. **C **VSL. **D** VCL. **E** VAP. **F** LIN. **G** STR. Data represent means ± SEM. * indicates *P* < 0.05, **P < 0.01 versus control. Two-way ANOVA analysis was performed

After the inhibition of DLD protein in HF and LF spermatozoa, it was found that the percent total motility of the HF bull spermatozoa was significantly decreased in comparison to the LF bull spermatozoa. The velocity parameters of the treated spermatozoa such as VCL, VSL and VAP significantly decreased in HF comparison with LF. Other CASA parameters BCF, STR, and LIN were also found to be decreased in HF spermatozoa with respect to LF spermatozoa (Fig. 7A–G). The mean of the sperm kinematic parameters is presented in Table S2.

## Discussion

The present work was undertaken to find out whether the spermatozoa from bulls of contrasting fertility differ in their DLD protein abundance and how this variation affects the ability of high- and low-fertile bull spermatozoa to fertilise. Sperm from bulls of contrasting fertility possess differential abundance of DLD, affecting the physiology of the sperm which in turn, modulates the fertilizing ability of the spermatozoa.

Dihydrolipoamide dehydrogenase or lipoamide dehydrogenase is a post-pyruvate-lactate enzyme [30]. It is component of pyruvate dehydrogenase complex (PDHc) [30]. PDHc is a multienzyme complex consisting of pyruvate dehydrogenase (E1, now renamed PDHA), dihydrolipoamide acetyltransferase (E2, now renamed DLAT), and lipoamide dehydrogenase (E3, now renamed DLD). Pyruvate dehydrogenase complex is canonically located in the mitochondrial matrix and catalyse the oxidative decarboxylation of pyruvate to acetyl CoA, the reduced form of nicotinamide adenine dinucleotide (NADH) and CO_2_, through a series of five reactions involving PDHA, DLAT, and DLD [30, 31]. DLD is a crucial enzyme in pyruvate metabolism as it forms a part of the pyruvate dehydrogenase complex, which metabolizes pyruvate directly.

The major source of ROS is due to pyruvate-lactate metabolism [32]. During metabolism DLD produces optimum amount of ROS, influences capacitation in spermatozoa is attributed to the ability to increase cAMP levels, which is required for the cAMP-dependent protein tyrosine phosphorylation, or its ability to influence calcium levels associated with capacitation of spermatozoa [33–35]. The optimum amount of ROS required to induce the capacitation during fertilization process. When ROS maintained at low or moderate concentrations, they play several beneficial roles. As discussed above ROS is crucial for induction of capacitation which regulates the ability of sperm to reach the site of fertilization and interact with egg.

This is the first-ever report on effect of presence of the higher abundance of ROS producing DLD protein in buffalo bull spermatozoa. In this report, it was found that ROS producing DLD protein was higher in LF bull in comparison to the HF bull spermatozoa. These results were corroborated with our previous study in which the DLD abundance were confirmed by Western blot in high and low fertile bull spermatozoa [21]. As it is partially known that DLD induced capacitation by producing ROS. What remains unclear whether higher abundance of DLD influences the fertilizing capacity of the low fertile.

To determine impact of higher DLD abundance in buffalo bull spermatozoa, we used specific inhibitor of DLD, i.e., 2-methoxyindole-5-carboxylic acid (MICA). MICA treatment was given in various contexts. We designed three experiments, conducted in capacitated medium (Fig. 1B); ROS level, sperm motility and acrosome reaction were assessed in spermatozoa after treatment with MICA.

DLD inhibition was confirmed by quantifying differential ROS level after MICA treatment [18, 25]. The cascading effect of inhibitor may limit the ROS production level, which in turn, results in a reduction in cAMP levels and, eventually, a decline in capacitation, hyperactivation as well as acrosome reaction (Fig. 1). The findings of this assay showed a considerably reduction in ROS generation after MICA treatment in time and concentration dependent manner. ROS production in the 5 mmol/L concentration at 0 h does not show any ROS inhibition, contrary at this concentration ROS production increase significantly (Fig. 4A and B). Same condition was observed at 0 h in the acrosome reaction levels at 5 mmol/L concentration. This could be due to, transfer of spermatozoa from the non-capacitated medium (NCM) to the capacitated medium at 0 h which might have affected the ROS and acrosome reaction inducing pathways in an unknown way, during the transition period of spermatozoa. Hence, exhibiting higher ROS production and acrosome reaction in comparison to the control samples. In this study the maximum decrease in the ROS production was observed in the 10 mmol/L concentration in all the time intervals. However, at 10 mmol/L concentration, a decrease in ROS production level was observed in all the time intervals, indicating complete saturation of the binding sites of DLD for MICA. Acrosome reaction after 10 mmol/L MICA treatment at every time interval shows significant decrease in acrosome reacted spermatozoa. However, these results were not constant with the other concentrations such as 2 h at 5 mmol/L concentration and 3 h for 1 mmol/L, 2.5 mmol/L and 5 mmol/L concentration exhibiting increase in the acrosome reaction. The reduction in the inhibition of acrosome reaction at these concentrations indicates the availability of MICA binding sites on DLD protein. The incubation for 4 h using all four concentrations of MICA followed a trend of significantly decreasing the levels of ROS production and acrosome reaction. Due to the aforementioned reasons, 10 mmol/L at 4 h were selected for the further experimentations. Previous finding also supports that DLD generates optimum amount of the ROS (such as peroxides and super-oxides) [36], which in turn have been directly implicated in sperm capacitation and acrosome reaction [33, 36–39]. Capacitation are series of changes in sperm’s structural and biochemical composition in such a way that they develop increased motility and are prepared for interactions with the egg (or oocyte) at fertilization site in FRT [40]. Our results further indicate that DLD is critical for hyperactivation during capacitation, as well as for the acrosome reaction, a post-capacitation event, as MICA, a particular DLD inhibitor, had an impact on both processes. These findings convincingly prove that the hypermotility and acrosome reaction directly depends on DLD activity in buffalo spermatozoa. Previously, Siva et al. [24] reported that hamster spermatozoal DLD is necessary for hyper motility during capacitation as well as the acrosome response, a post-capacitation event. More importantly, we show that DLD protein localized in the spermatozoa’s head and in middle piece of spermatozoa and the quantity of DLD was more pronounced in the head region of LF bull spermatozoa with respect to the HF bulls (Fig. 3) indicating an increased production of ROS in LF bull spermatozoa. These evidences are in support of the earlier reports, wherein they highlighted that PDHA and DLD were present in extramitochondrial sites and these extramitochondrial components generated ROS in hamster spermatozoa supporting their functional abilities [18, 19, 25, 32].

Although, we established the involvement of DLD protein in ROS generation, capacitation, and acrosome response, still the question remains unsolved, firstly, what happens to sperm that contains an excessive amount of DLD and secondly, how it impacts the fertilizing potential of spermatozoa. To investigate these queries, we provide evidence that an excessive amount of the DLD protein in spermatozoa causes detrimental effect leading to early capacitation, which reduces the fertilizing potential of spermatozoa. Our study reported the reduced ROS production and acrosome reaction in control (capacitated) HF spermatozoa, in comparison to the LF spermatozoa. This finding relates to the lower abundance of DLD protein in the HF group in comparison to its higher abundance in the LF group further producing increased ROS levels along with higher rate of acrosome reaction (Fig. 6A and B). To validate this hypothesis further, MICA treatment showed significant inhibition of DLD protein in HF spermatozoa as the initial abundance of this protein was low, so 10 mmol/L concentration of MICA at 4 h fully saturated the protein which further showed inhibitory effect by reducing the ROS production and rate of acrosome reaction. Whereas, the initial abundance of DLD was significantly higher in the LF spermatozoa, so the treatment using 10 mmol/L at 4 h of MICA did not significantly influence the DLD activity, hence showed the increase in both ROS levels and the acrosome reaction. Taken altogether, differential abundance of DLD protein in high and low fertile bull exhibit differential production of ROS in spermatozoa. Furthermore, in low fertile bulls, higher level of DLD may lead to early capacitation, due to production of high abundance of ROS which eventually lowers the fertilizing ability of the bulls. In an earlier study, implication of the DLD protein in hamster spermatozoa was explained using MICA and BP inhibitors. They found that inhibition of DLD protein resulted in significant reduction of ROS production and concluded that hamster spermatozoa required DLD protein for ROS generation utilize for capacitation initiation [32]. Simultaneously, it was also observed that DLD inhibition reduced the hyperactivation and acrosome reaction in HF bulls in comparison to the low fertile bull spermatozoa (Figs. 6B and 7). This could be due to production of the ROS level after DLD inhibition. In HF group, most the protein actively inhibits by the MICA, which shows a strong reduction in ROS production and ultimately decreased kinematic parameters and AR. Concordantly, in a previous study, hamster spermatozoa was assessed for significance of the DLD protein and they found that DLD is necessary for the hamster acrosome reaction [32]. Altogether, our investigation highlights the higher abundance of DLD protein in the spermatozoa produces large amount of ROS which causes the early hypermotility or capacitation and acrosome reaction in the buffalo spermatozoa. This condition may reduce the fertilizing ability of the spermatozoa in female reproductive tract. Thus, the study provides a clear insight into detrimental effect due to increased abundance of the DLD protein in spermatozoa as well as indicates that the optimum amount of DLD is required to perform fertilization process in the female reproductive tract.

## Conclusion

The compelling evidences of this study highlights that low-fertility buffalo bull spermatozoa contain higher abundances of DLD protein as compared to high-fertility bull spermatozoa. This increased DLD abundance leads to elevated ROS production in low-fertility spermatozoa, resulting in early capacitation and a reduced fertilizing potential. Altogether, the findings support that DLD protein regulates the fertilizing capacity of sperm through the modulation of vital sperm functions, i.e., capacitation, hyperactivation, and the acrosome reaction.

## Supplementary Information

**Additional file 1: Fig. S1.** Graph representing the optimum amount of ROS produced by non-capacitated spermatozoa. **Fig. S2.** Graph representing the amount of ROS and acrosome reaction produced in controland MICA treated spermatozoa in high and low fertile bulls. **Fig. S3.** Graph representing CFDA-PI or live dead staining of MICA treated spermatozoa. **Table S1.** Dose and time-dependent effect of MICA on motility of buffalo spermatozoa. **Table S2.** Dose and time-dependent effect of MICA on motility of HF and LF buffalo spermatozoa.

## Data Availability

The datasets used and/or analysed during the current study are available from the corresponding author on reasonable request.

## Abbreviations

AR: Acrosome reaction
BCF: Beat-cross frequency
BSA: Bovine serum albumin
CASA: Computer assisted semen analysis
CR: Conception rate
CTC: Chlortetracycline
DLD: Dihydrolipoamide dehydrogenase
FITC-PNA: Fluorescein isothiocyanate-conjugated peanut agglutinin
FRT: Female reproductive tract
HF: High fertile
LF: Low fertile
LIN: Linearity
MCT: Microcentrifuge tube
MFI: Mean fluorescence intensity
MICA: 2-Methoxyindole-5-carboxylic acid
NCM: Non-capacitated medium
ROI: Region of interest
ROS: Reactive oxygen species
STR: Straightness
TM: Total motility
VAP: Average path velocity
VCL: Curvilinear velocity
VSL: Straight-line velocity

## References

[1] Kashir J, Heindryckx B, Jones C, De Sutter P, Parrington J, Coward K. Oocyte activation, phospholipase C zeta and human infertility. Hum Reprod Update. 2010;16(6):690–703. 20573804 10.1093/humupd/dmq018

[2] Austin CR. Observations on the penetration of the sperm in the mammalian egg. Aust J Biol Sci. 1951;4(4):581–96. 10.1071/BI9510581 14895481

[3] Jha KN, Kameshwari DB, Shivaji S. Role of signaling pathways in regulating the capacitation of mammalian spermatozoa. Cell Mol Biol (Noisy-le-grand). 2003;49:329–40. 12887085

[4] Kulanand J, Shivaji S. Capacitation-associated changes in protein tyrosine phosphorylation, hyperactivation and acrosome reaction in hamster spermatozoa. Andrologia. 2001;33(2):95–104. 11350373 10.1046/j.1439-0272.2001.00410.x

[5] Visconti PE, Kopf GS. Regulation of protein phosphorylation during sperm capacitation. Biol Reprod. 1998;59(1):1–6. 9674985 10.1095/biolreprod59.1.1

[6] Visconti PE, Moore GD, Bailey JL, Leclerc P, Connors SA, Pan D, et al. Capacitation of mouse spermatozoa. II. Protein tyrosine phosphorylation and capacitation are regulated by a cAMP-dependent pathway. Development. 1995;121(4):1139–50. 7538069 10.1242/dev.121.4.1139

[7] Yanagimachi R. Fertility of mammalian spermatozoa: Its development and relativity. Zygote. 1994;2(4):371–2. 8665172 10.1017/S0967199400002240

[8] Flesch FM, Colenbrander B, Van Golde LMG, Gadella BM. Capacitation induces tyrosine phosphorylation of proteins in the boar sperm plasma membrane. Biochem Biophys Res Commun. 1999;262(3):787–92. 10471403 10.1006/bbrc.1999.1300

[9] Osheroff JE, Visconti PE, Valenzuela JP, Travis AJ, Alvarez J, Kopf GS. Regulation of human sperm capacitation by a cholesterol efflux- stimulated signal transduction pathway leading to protein kinase A-mediated up-regulation of protein tyrosine phosphorylation. Mol Hum Reprod. 1999;5(11):1017–26. 10541563 10.1093/molehr/5.11.1017

[10] Pukazhenthi BS, Long JA, Wildt DE, Ottinger MA, Armstrong DL, Howard J. Regulation of sperm function by protein tyrosine phosphorylation in diverse wild felid species. J Androl. 1998;19(6):675–85. 9876019 10.1002/j.1939-4640.1998.tb02077.x

[11] Si Y, Okuno M. Role of tyrosine phosphorylation of flagellar proteins in hamster sperm hyperactivation. Biol Reprod. 1999;61(1):240–6. 10377055 10.1095/biolreprod61.1.240

[12] Carrera A, Moos J, Ning XP, Gerton GL, Tesarik J, Kopf GS, et al. Regulation of protein tyrosine phosphorylation in human sperm by a calcium/calmodulin-dependent mechanism: Identification of A Kinase Anchor Proteins as major substrates for tyrosine phosphorylation. Dev Biol. 1996;180(1):284–96. 8948591 10.1006/dbio.1996.0301

[13] Johnson LR, Foster JA, Haig-Ladewig L, Vanscoy H, Rubin CS, Moss SB, et al. Assembly of AKAP82, a protein kinase a anchor protein, into the fibrous sheath of mouse sperm. Dev Biol. 1997;192(2):340–50. 9441672 10.1006/dbio.1997.8767

[14] Brito M, Figueroa J, Maldonado EU, Vera JC, Burzio LO. The major component of the rat sperm fibrous sheath is a phosphoprotein. Gamete Res. 1989;22(2):205–17. 2707727 10.1002/mrd.1120220208

[15] Jha KN, Shivaji S. Identification of the major tyrosine phosphorylated protein of capacitated hamster spermatozoa as a homologue of mammalian sperm a kinase anchoring protein. Mol Reprod Dev. 2002;61(2):258–70. 11803562 10.1002/mrd.1155

[16] Ecroyd H, Jones RC, Aitken RJ. Tyrosine phosphorylation of HSP-90 during mammalian sperm capacitation. Biol Reprod. 2003;69(6):1801–7. 12890735 10.1095/biolreprod.103.017350

[17] Naaby-Hansen S, Mandal A, Wolkowicz MJ, Sen B, Westbrook VA, Shetty J, et al. CABYR, a novel calcium-binding tyrosine phosphorylation-regulated fibrous sheath protein involved in capacitation. Dev Biol. 2002;242(2):236–54. 11820818 10.1006/dbio.2001.0527

[18] Mitra K, Rangaraj N, Shivaji S. Novelty of the pyruvate metabolic enzyme dihydrolipoamide dehydrogenase in spermatozoa: Correlation of its localization, tyrosine phosphorylation, and activity during sperm capacitation. J Biol Chem. 2005;280(27):25743–53. 15888450 10.1074/jbc.M500310200

[19] Kumar V, Rangaraj N, Shivaji S. Activity of pyruvate dehydrogenase A (PDHA) in hamster spermatozoa correlates positively with hyperactivation and is associated with sperm capacitation. Biol Reprod. 2006;75(5):767–77. 16855207 10.1095/biolreprod.106.053587

[20] Breitbart H, Rubinstein S, Lax Y. Regulatory mechanisms in acrosomal exocytosis. Rev Reprod. 1997;1(2):165–74. 10.1530/ror.0.0020165 9414480

[21] Karanwal S, Pal A, Chera JS, Batra V, Kumaresan A, Datta TK, et al. Identification of protein candidates in spermatozoa of water buffalo (*Bubalus bubalis*) bulls helps in predicting their fertility status. Front Cell Dev Biol. 2023;11:1119220. 10.3389/fcell.2023.1119220 PMC9986327 36891514

[22] Kim BJ, Park DR, Nam TS, Lee SH, Kim UH. Seminal CD38 enhances human sperm capacitation through its interaction with CD31. PLoS One. 2015;10(9):e0139110. 26407101 10.1371/journal.pone.0139110 PMC4583300

[23] Panneerdoss S, Siva AB, Kameshwari DB, Rangaraj N, Shivaji S. Association of lactate, intracellular pH, and intracellular calcium during capacitation and acrosome reaction: Contribution of hamster sperm dihydrolipoamide dehydrogenase, the E3 subunit of pyruvate dehydrogenase complex. J Androl. 2012;33(4):699–710. 21903972 10.2164/jandrol.111.013151

[24] Siva AB, Panneerdoss S, Sailasree P, Singh DK, Kameshwari DB, Shivaji S. Inhibiting sperm pyruvate dehydrogenase complex and its E3 subunit, dihydrolipoamide dehydrogenase affects fertilization in Syrian hamsters. PLoS One. 2014;9(5):e97916. 24852961 10.1371/journal.pone.0097916 PMC4031208

[25] Mitra K, Shivaji S. Novel tyrosine-phosphorylated post-pyruvate metabolic enzyme, Dihydrolipoamide dehydrogenase, involved in capacitation of hamster spermatozoa. Biol Reprod. 2004;70(4):887–99. 14645106 10.1095/biolreprod.103.022780

[26] Yang X, Song J, Yan LJ. Chronic inhibition of mitochondrial dihydrolipoamide dehydrogenase (DLDH) as an approach to managing diabetic oxidative stress. Antioxidants. 2019;8(2):32. 30717346 10.3390/antiox8020032 PMC6406859

[27] Ambrus A, Adam-vizi V. Neurochemistry International Human dihydrolipoamide dehydrogenase (E3) deficiency : novel insights into the structural basis and molecular pathomechanism. Neurochem Int. 2018;1(117):5–14. 10.1016/j.neuint.2017.05.018 28579060

[28] Aparicio IM, Gil MC, Garcia-Herreros M, Peña FJ, Garcia-Marin LJ. Inhibition of phosphatidylinositol 3-kinase modifies boar sperm motion parameters. Reproduction. 2005;129(3):283–9. 15749955 10.1530/rep.1.00447

[29] Singh I, Balhara AK. New approaches in buffalo artificial insemination programs with special reference to India. Theriogenology. 2016;86(1):194–9. 27155729 10.1016/j.theriogenology.2016.04.031

[30] Patel MS, Vettakkorumakankav NN, Liu TC. Dihydrolipoamide dehydrogenase: Activity assays. Methods Enzymol. 1995;252:186–95. 7476353 10.1016/0076-6879(95)52022-8

[31] Reed LJ. Regulation of mammalian pyruvate dehydrogenase complex by a phosphorylation-Dephosphorylation cycle. Curr Top Cell Regul. 1981;18:95–106. 7273851 10.1016/B978-0-12-152818-8.50012-8

[32] Kumar V, Kota V, Shivaji S. Hamster sperm capacitation: Role of pyruvate dehydrogenase A and dihydrolipoamide dehydrogenase. Biol Reprod. 2008;79(2):190–9. 18401010 10.1095/biolreprod.107.066704

[33] Zhou ZH, McCarthy DB, O’Connor CM, Reed LJ, Stoops JK. The remarkable structural and functional organization of the eukaryotic pyruvate dehydrogenase complexes. Proc Natl Acad Sci. 2001;98(26):14802–7. 11752427 10.1073/pnas.011597698 PMC64939

[34] Rivlin J, Mendel J, Rubinstein S, Etkovitz N, Breitbart H. Role of hydrogen peroxide in sperm capacitation and acrosome reaction. Biol Reprod. 2004;70(2):518–22. 14561655 10.1095/biolreprod.103.020487

[35] O’Flaherty C, Breininger E, Beorlegui N, Beconi MT. Acrosome reaction in bovine spermatozoa: Role of reactive oxygen species and lactate dehydrogenase C4. Biochim Biophys Acta. 2005;1726(1):96–101. 16112812 10.1016/j.bbagen.2005.07.012

[36] Starkov AA, Fiskum G, Chinopoulos C, Lorenzo BJ, Browne SE, Patel MS, et al. Mitochondrial α-ketoglutarate dehydrogenase complex generates reactive oxygen species. J Neurosci. 2004;24(36):7779–88. 15356189 10.1523/JNEUROSCI.1899-04.2004 PMC6729932

[37] Aitken RJ, Paterson M, Fisher H, Buckingham DW, Van Duin M. Redox regulation of tyrosine phosphorylation in human spermatozoa and its role in the control of human sperm function. J Cell Sci. 1995;108(5):2017–25. 7544800 10.1242/jcs.108.5.2017

[38] De Lamirande E, Leclerc P, Gagnon C. Capacitation as a regulatory event that primes spermatozoa for the acrosome reaction and fertilization. Mol Hum Reprod. 1997;3(3):175–94. 9237244 10.1093/molehr/3.3.175

[39] Aitken R. Molecular mechanisms regulating human sperm function. Mol Hum Reprod. 1997;3(3):169–73. 9237243 10.1093/molehr/3.3.169

[40] Vernet P, Fulton N, Wallace C, Aitken RJ. Analysis of reactive oxygen species generating systems in rat epididymal spermatozoa. Biol Reprod. 2001;65(4):1102–13. 11566731 10.1095/biolreprod65.4.1102

