# Supplementary material for: Higher abundance of DLD protein in buffalo bull spermatozoa causes elevated ROS production leading to early sperm capacitation and reduction in fertilizing ability
Source: J Anim Sci Biotechnol. 2024 Sep 11;15:126. doi: 10.1186/s40104-024-01085-6 (PMC11389063; doi:10.1186/s40104-024-01085-6)
Supplement: Supplementary file 1 — Additional file 1: Fig. S1. Graph representing the optimum amount of ROS produced by non-capacitated spermatozoa. Fig. S2. Graph representing the amount of ROS and acrosome reaction produced in controland MICA treated spermatozoa in high and low fertile bulls. Fig. S3. Graph representing CFDA-PI or live dead staining of MICA treated spermatozoa. Table S1. Dose and time-dependent effect of MICA on motility of buffalo spermatozoa. Table S2. Dose and time-dependent effect of MICA on motility of HF and LF buffalo spermatozoa. [file 40104_2024_1085_MOESM1_ESM.docx]

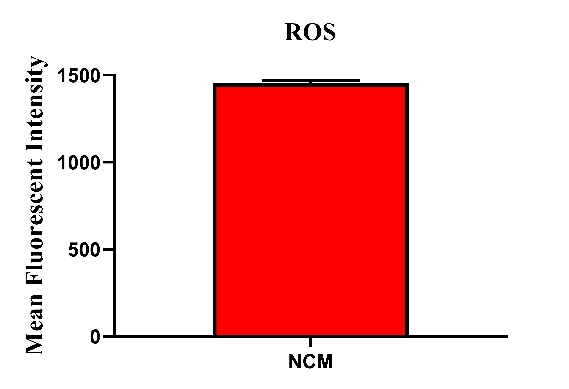


**Fig. S1** Graph representing the optimum amount of ROS produced by non-capacitated spermatozoa in non-capacitating medium


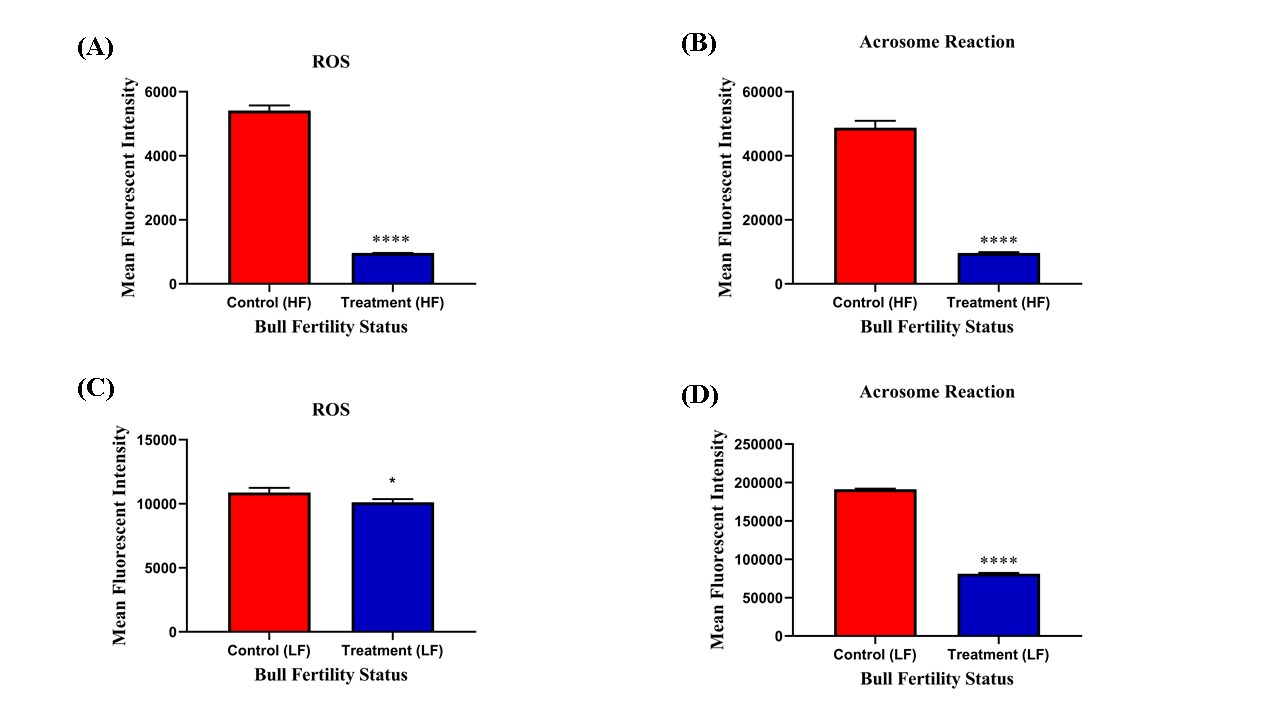


**Fig. S2** Graph representing the amount of ROS and acrosome reaction produced in control (capacitated) and MICA treated spermatozoa in high and low fertile bulls. **(A)** Average MFI histograms of ROS produced in control (capacitated) HF and MICA treated HF bull spermatozoa. **(B)** Average MFI histograms of acrosome reaction in control (capacitated) HF and MICA treated HF bull spermatozoa. **(C)** Average MFI histograms of ROS produced in control (capacitated) LF and MICA treated LF bull spermatozoa. **(D)** Average MFI histograms of acrosome reaction in control (capacitated) LF and MICA treated LF bull spermatozoa


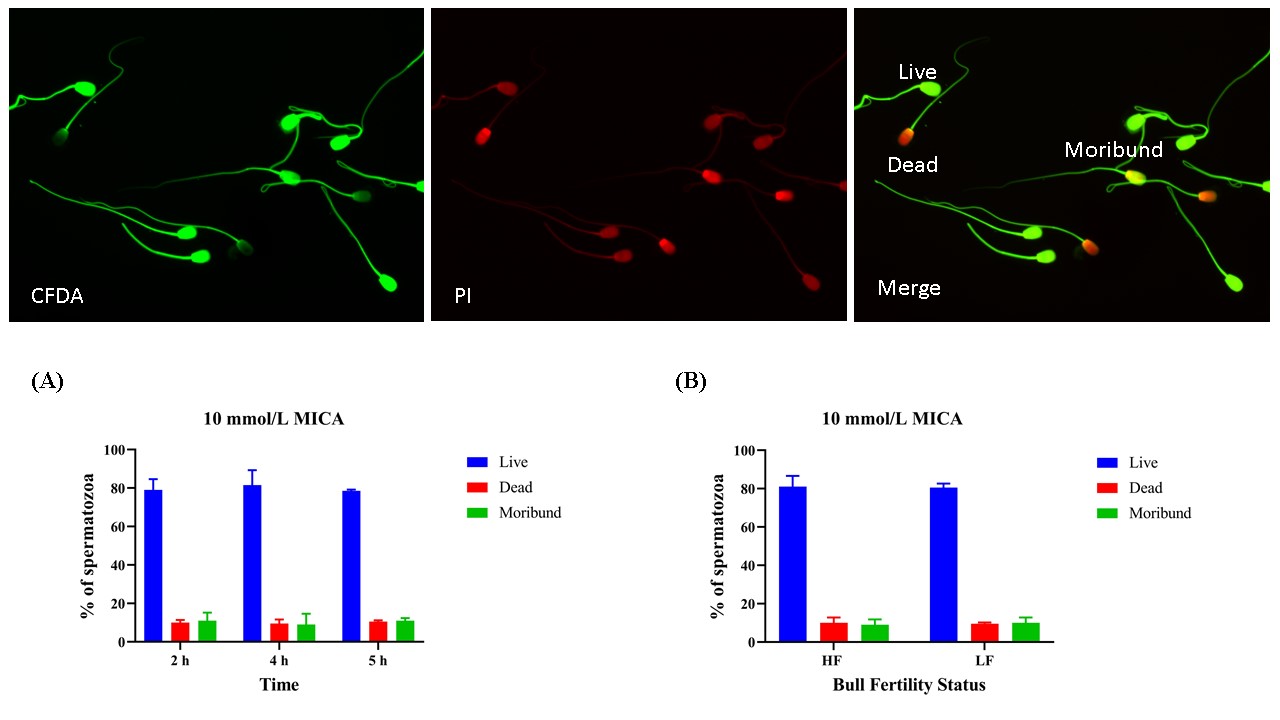


**Fig. S3** Graph representing CFDA-PI or live dead staining of MICA treated spermatozoa. The intense green fluorescence could be seen in sperms with their membranes intact, but bright red fluorescence was seen in spermatozoa that were dead. Moribund spermatozoa fluoresced in a combination of red and green giving an orange colour. In live-dead staining, after MICA treatment at 2 h, 4 h and 5 h in the maximum concentration i.e., 10 mmol/L was performed to check the toxicity of the MICA. In this assay no significant difference was not observed among different time interval at 10 mmol/L MICA concentration. Live and dead spermatozoa were also checked in the HF and LF bulls after MICA treatment but did not show any significant difference

**Table S1** Dose and time-dependent effect of MICA on motility of buffalo spermatozoa

| **TM** | | | | | |
| --- | --- | --- | --- | --- | --- |
|  | **Control** | **1 mmol/L** | **2.5 mmol/L** | **5 mmol/L** | **10 mmol/L** |
| 0 h | 66.4 ± 0.8 | 74 ± 1.7 | 68 ± 6.3 | 72.8 ± 0.8* | 63 ± 10** |
| 1 h | 65.8 ± 2.8 | 40.7 ± 2.5 | 45.6 ± 1.8 | 64.4 ± 3.4 | 55.8 ± 13.2** |
| 2 h | 74.8 ± 1.7 | 79.2 ± 1 | 90.7 ± 4.2 | 78.1 ± 0.7 | 79.2 ± 7.2** |
| 3 h | 72.3 ± 5.9 | 84.2 ± 7.2 | 67.8 ± 5 | 81.4 ± 2.6* | 47.4 ± 4.8*** |
| 4 h | 75.3 ± 5.2 | 75.6 ± 1.7 | 48.7 ± 52.6* | 87.1 ± 0.8*** | 55.3 ± 2.5**** |
| 5 h | 79.6 ± 1.5 | 79.6 ± 8.3 | 87.1 ± 0.9 | 87.9 ± 4.7** | 64.1 ± 13.9** |
| **BCF** | | | | | |
|  | **Control** | **1 mmol/L** | **2.5 mmol/L** | **5 mmol/L** | **10 mmol/L** |
| 0 h | 29.9 ± 1.2 | 29.1 ± 1.1 | 27.9 ± 1.7 | 27.3 ± 1.7* | 25.3 ± 2*** |
| 1 h | 27.4 ± 0 | 26.6 ± 0.6 | 26.8 ± 0.5 | 24.7 ± 0.1* | 22.6 ± 0.4**** |
| 2 h | 28.5 ± 0.2 | 28.4 ± 0.5 | 26.6 ± 1* | 26.8 ± 1.3** | 25.5 ± 1*** |
| 3 h | 27.9 ± 0.1 | 27.3 ± 0.1 | 26.5 ± 0.9* | 26.9 ± 0.5** | 26.4 ± 0.7*** |
| 4 h | 28 ± 0 | 27.6 ± 0.3 | 30.8 ± 6.1** | 26.1 ± 0**** | 24.8 ± 0 |
| 5 h | 29.3 ± 0.2 | 28.4 ± 0.4 | 26.7 ± 0.2* | 27 ± 0.5 | 24.2 ± 0.3 |
| **VAP** | | | | | |
|  | **Control** | **1 mmol/L** | **2.5 mmol/L** | **5 mmol/L** | **10 mmol/L** |
| 0 h | 66.4 ± 10.5 | 72.7 ± 12.4 | 63.4 ± 18.5 | 72 ± 17.8 | 67.4 ± 17.8 |
| 1 h | 53.3 ± 12.2 | 63.5 ± 1.8 | 60.5 ± 1.1 | 63 ± 11.1 | 62 ± 3.3 |
| 2 h | 50.3 ± 10.5 | 53.7 ± 7.2 | 46.5 ± 7.9 | 41.7 ± 0.1 | 39.2 ± 0.7 |
| 3 h | 45 ± 0.6 | 48.2 ± 4.9 | 35.6 ± 3.1 | 32.9 ± 5.3 | 39.7 ± 6.4 |
| 4 h | 50.8 ± 9.3 | 44.6 ± 3.2 | 54.6 ± 1.6 | 41.2 ± 7.3 | 44.9 ± 10.7 |
| 5 h | 48.1 ± 14 | 42.2 ± 2.2 | 38.5 ± 4.8 | 35 ± 3.9 | 30.4 ± 3.7 |
| **LIN** | | | | | |
|  | **Control** | **1 mmol/L** | **2.5 mmol/L** | **5 mmol/L** | **10 mmol/L** |
| 0 h | 55.9 ± 5.1 | 54.4 ± 2.7 | 56.9 ± 7 | 46.5 ± 11.4 | 48.1 ± 20.4 |
| 1 h | 54.5 ± 6.2 | 44.7 ± 4.1 | 49.7 ± 1.3 | 41.9 ± 7.1 | 50.1 ± 16.6 |
| 2 h | 65.7 ± 3.4 | 64.4 ± 1.6 | 62.3 ± 6 | 65.6 ± 1.7 | 70.5 ± 1.7 |
| 3 h | 63.2 ± 5.7 | 69.8 ± 5.7 | 63.8 ± 4.3 | 75 ± 0.4 | 60.8 ± 6.5** |
| 4 h | 62.3 ± 3.9 | 68.4 ± 4.2 | 53 ± 24 | 66.3 ± 3.6 | 59 ± 9.5** |
| 5 h | 62.5 ± 8.4 | 65.5 ± 9.1 | 72.4 ± 6.2 | 75.5 ± 0.6 | 64.2 ± 6.9* |
| **VCL** | | | | | |
|  | **Control** | **1 mmol/L** | **2.5 mmol/L** | **5 mmol/L** | **10 mmol/L** |
| 0 h | 118.5 ± 1.4 | 133 ± 2.8 | 116.5 ± 3.8 | 138.1 ± 4.7 | 126.1 ± 5.2 |
| 1 h | 96.6 ± 3.5 | 130.8 ± 5.6 | 113.7 ± 5.8 | 136 ± 23.4 | 118.8 ± 7.2 |
| 2 h | 88 ± 3.8 | 93.9 ± 1.5 | 86.7 ± 1.5* | 72 ± 2.1* | 64.6 ± 4.3* |
| 3 h | 75.3 ± 2.9 | 78.3 ± 12.3 | 69 ± 10* | 52.9 ± 8.1** | 71.6 ± 3* |
| 4 h | 88.5 ± 5.1 | 72.5 ± 1.2 | 103.6 ± 4.7* | 72.1 ± 11.3** | 75.8 ± 2.2** |
| 5 h | 91.6 ± 4.4 | 78.1 ± 14.4 | 64.4 ± 16.6 | 54.6 ± 5.1** | 56.8 ± 13.4** |
| **VSL** | | | | | |
|  | **Control** | **1 mmol/L** | **2.5 mmol/L** | **5 mmol/L** | **10 mmol/L** |
| 0 h | 49 ± 6.6 | 52.2 ± 6.6 | 46.2 ± 10.8 | 51.1 ± 12.2 | 45.3 ± 6.6 |
| 1 h | 37.2 ± 7.3 | 42.5 ± 0.1 | 40.4 ± 0.7 | 38 ± 3.6 | 39.7 ± 11.6 |
| 2 h | 37.8 ± 6 | 39.6 ± 4.9 | 32.5 ± 4.2 | 30.3 ± 0.2* | 29.8 ± 0.2* |
| 3 h | 32.3 ± 2.7 | 36.4 ± 2.9 | 25.1 ± 2.6 | 26.1 ± 4.4* | 28.3 ± 5.9** |
| 4 h | 37.2 ± 5.7 | 32.8 ± 0.1 | 37.2 ± 6.8 | 30.3 ± 4.5 | 31.9 ± 4.8** |
| 5 h | 34.5 ± 6.8 | 30.9 ± 0.1 | 29.3 ± 2.4 | 28.5 ± 3.7 | 22.9 ± 1.3** |
| **STR** | | | | | |
|  | **Control** | **1mmol/L** | **2.5mmol/L** | **5mmol/L** | **10mmol/L** |
| 0 h | 81.1 ± 3.1 | 80.2 ± 1.7 | 82.7 ± 6.2 | 76.7 ± 5 | 73.9 ± 10.7 |
| 1 h | 79 ± 3.6 | 74.7 ± 2 | 75.4 ± 0.3 | 70.4 ± 6.4 | 75.1 ± 10.2 |
| 2 h | 84.9 ± 2.6 | 83.3 ± 3.4 | 82.2 ± 3.2 | 84 ± 1 | 85.9 ± 0.3 |
| 3 h | 81.9 ± 4.1 | 85.7 ± 2.6 | 84.8 ± 2 | 89.7 ± 0.8 | 82 ± 2.7 |
| 4 h | 82.9 ± 3.1 | 85.1 ± 4.7 | 77.7 ± 13.9 | 84.6 ± 3.9 | 80 ± 6.3 |
| 5 h | 83.8 ± 6.7 | 85.3 ± 3.2 | 87.4 ± 1.9 | 89 ± 0.4 | 85.2 ± 4.1 |

Percentage sperm kinetic parameter were evaluated by CASA in spermatozoa. MICA used in different concentrations i.e., 1 mmol/L, 2.5 mmol/L, 5 mmol/L, and 10 mmol/L were adjusted throughout a variety of time intervals i.e. 0 h, 1 h, 2 h, 3 h, 4 h, and 5 h. Data represent means ± SEM. **P* < 0.05, ***P* < 0.01, ****P* < 0.001 and *****P* < 0.0001 versus respective control, ANOVA analysis was performed

**Table S2** Dose and time-dependent effect of MICA on motility of HF and LF buffalo spermatozoa

|  |  | **HF** | **LF** |
| --- | --- | --- | --- |
| 1. | **BCF** | 18.8 ± 1.2 | 22.5 ± 1.06 |
| 2. | **VSL** | 22.91 ± 1.6 | 26.1 ± 1.3 |
| 3. | **VCL** | 70.2 ± 3.02 | 82.09 ± 2.9 |
| 4. | **LIN** | 44.3 ± 4.5 | 55.6 ± 2.4 |
| 5. | **VAP** | 38.1 ± 2.6 | 43.61 ± 1.6 |
| 6. | **TM** | 44.9 ± 2.7 | 54.4 ± 3.6 |
| 7. | **STR** | 77.3 ± 1.7 | 84.9 ± 2.5 |

Percentage sperm kinetic parameter were evaluated by CASA in spermatozoa. MICA used in different concentrations i.e.,1 mmol/L, 2.5 mmol/L, 5 mmol/L, and 10 mmol/L were adjusted throughout a variety of time intervals i.e., 0 h, 1 h, 2 h, 3 h, 4 h, and 5 h. Data represent means ± SEM. **P* < 0.05, ***P* < 0.01, ****P* < 0.001 and *****P* < 0.0001 versus respective control, ANOVA analysis was performed.
